# Effects of compound lactic acid bacteria on the quality and microbial diversity of alfalfa silage in saline-alkali soils

**DOI:** 10.3389/fmicb.2024.1524296

**Published:** 2025-03-06

**Authors:** Si-Yi Wang, Le Sun, Heng Jiang, Guo-Lin Yang, Zhen-Nan He, Yuan-Yuan Jing, Feng-Qin Gao

**Affiliations:** ^1^College of Grassland Science, Qingdao Agricultural University, Qingdao, China; ^2^Institute of Grassland Research, Chinese Academy of Agricultural Science, Hohhot, China

**Keywords:** saline-alkali alfalfa, compound lactic acid bacteria, silage quality, microbial diversity, feed

## Abstract

With the rapid development of animal husbandry, forage resources are increasingly scarce. Improving the utilization rate of forage products and silage efficiency of planting is an urgent problem to be solved. This experiment used high moisture alfalfa at the budding stage with a water content of 71.4% from saline-alkali and non-saline-alkali soils as raw materials, setting up four experimental groups: non-saline-alkali alfalfa without additives (HNS-CK), non-saline-alkali alfalfa with compound lactic acid bacteria (*Lactobacillus* plantarum + *Lactobacillus* buchneri + *Pediococcus* pentosaceus + *Lactobacillus* kimchi, HNS-L4), saline-alkali alfalfa without additives (HS-CK), and saline-alkali alfalfa with compound lactic acid bacteria (HS-L4). After 60 days of silage, the quality and microbial diversity of the silage were tested. The results showed that the dry matter (DM) and lactic acid (LA) of the HNS-L4 group were significantly higher than those of the HS-L4 (*P* < 0.05), while there was no significant difference in crude protein (CP) between the HNS-L4 group and the HS-L4 (*P* < 0.05). The neutral detergent fiber (NDF), acid detergent fiber (ADF), pH of the HNS-L4 group were all lower than those of the HS-L4. The results of the microbial community showed that compared with the non-additives group, the Shannon index decreased and the Simpson index increased in the compound lactic acid bacteria group, indicating a significant reduction in microbial diversity in the silage environment (*P* < 0.05). The dominant bacteria in the HNS-CK and HS-CK groups were *Enterobacteriaceae*, while the dominant bacteria in the HNS-L4 and HS-L4 groups were *Lactobacillus*. At the phylum level, the dominant bacteria in alfalfa after lactic acid bacteria treatment were Firmicutes, which were significantly higher than the control group (*P* < 0.05). Therefore, compound lactic acid bacteria can improve the quality of alfalfa silage in both saline alkali and non-saline-alkali soils, with saline-alkali soils being better than non-saline-alkali soils, and both can reduce microbial diversity.

## Introduction

In recent years, Chinese animal husbandry has been continuously developing, especially the development of ruminant animal husbandry, which has led to adjustments in the structure of animal feed, with alfalfa occupying a significant share. According to the National Animal Husbandry Station, the demand for alfalfa as the preferred high-quality feed for animals in Chinese market is showing a trend of increasing year by year. The total planting area of alfalfa in China has reached 3.67 million hectares, but the actual planting area is far from meeting the needs of the development of animal husbandry. This determines that increasing the planting area and yield per unit area of alfalfa has become an urgent problem to be solved in actual production. However, Chinese cultivated area is insufficient to produce a sufficient amount of high-quality alfalfa. Under the policy of green-high quality-sustainable development, utilizing saline-alkali land resources is an important way to develop the alfalfa industry. Chinese saline-alkali land area is 99.13 million hectares, accounting for 1.03% of the national land area (Yang and Wang, 2015). The saline-alkali land is severely compacted, the improvement methods are lagging behind, which restricts the utilization of the land. Therefore, planting suitable plants on saline-alkali land can not only regulate the soil salinity content but also plays an irreplaceable role in improving saline-alkali land and preventing soil erosion (Chen et al., 2018).

It is alfalfa's rich nutritional content and high protein content that has attracted attention both domestically and internationally (Li and Wan, 2005; Radovc et al., 2009). Planting alfalfa in saline-alkali soils can not only improve the soil environment of saline-alkali soils but also serves as an excellent solution to the current problem of insufficient forage. In production, the main use of alfalfa is in the form of hay. During the preparation of hay, due to the plants own respiratory activity and external environmental factors such as leaf fall, exposure to rain causing spoilage, the loss rate of forage nutrients can reach up to 30% (Liu, 2018). High moisture alfalfa silage without being exposed to the ground can effectively avoid loss caused by the rainy season and is more suitable for the production of feed in areas with abundant rainfall. However, alfalfa has low soluble carbohydrates and high buffering capacity, making single silage difficult to succeed (Saricicek and Kilic, 2011). In addition, when the moisture content of alfalfa is too high, harmful bacteria such as Enterobacteriaceae and Clostridium will proliferate rapidly. Clostridium not only decomposes proteins, reducing nutritional quality, but also competes with lactic acid bacteria for fermentation substrates (Liu et al., 2022; Bai et al., 2021; Zhao, 2019). At the beginning of silage, it is necessary to effectively control the proliferation of Clostridium to prevent the deterioration of silage feed (Li et al., 2020; Mu et al., 2020; Zhou, 2004). In response to the above problems with high moisture alfalfa single silage, this study explores the effect of improving silage quality by adding compound lactic acid bacteria. In current research, lactic acid bacteria, as the primary agent of fermentation, can significantly reduce the duration of silage during the fermentation process, suppress the proliferation of spoilage microorganisms, enhance palatability, and optimize the quality of silage (Li et al., 2021; Pahlow et al., 2003; Muck et al., 2018; Sousa et al., 2008; Zhong et al., 2017).

However, current research mainly focuses on the preparation of hay and introduction and selection in saline-alkali and non-saline-alkali soils, but there is little research on high moisture alfalfa silage in both saline-alkali and non-saline-alkali soils (Adesogan, 2008; Porcel et al., 2012; Wang et al., 2019; Nishiuchi et al., 2007; Si, 2012). This experiment selected compound lactic acid bacteria (*Lactobacillus* plantarum, *Lactobacillus* buchneri, *Pediococcus* pentosaceus, and *Lactobacillus* kimchi) that tolerate saline-alkali and non-saline-alkali soils, in order to provide a high-quality theoretical basis for the processing technology of high moisture alfalfa (Schmidt et al., 2009; Sun et al., 2014; Wan et al., 2021, 2007).

## Materials and methods

### Experimental materials

The alfalfa variety used in the experiment was Zhongmu No. 3, harvested on August 1^st^, 2023, at the second cutting of the budding stage at the Ba Yan Naoer City Agricultural and Animal Husbandry Science Research Institute Base 107°29E, 40°80N in the Inner Mongolia Autonomous Region. The average annual temperature at the base is 3.7°C–7.6°C, with a frost-free period of 126 days. The soil nutrient content in the 0–10 cm non-saline-alkali soil at the base is: pH value 8.40 (Wei et al., 2013), organic matter 18.00 g/kg, total nitrogen 0.92 g/kg, alkali-hydrolyzable nitrogen 102.00 mg/kg. The soil nutrient content in the saline-alkali soil is: pH value 9.25, organic matter 34.27 g/kg, total nitrogen 1.42 g/kg, and alkali-hydrolyzable nitrogen 117.39 mg/kg (Xiao et al., 2019).

The experiment selected four types of lactic acid bacteria, *Lactobacillus* plantarum 1 × 10^9^ cfu/g, *Lactobacillus* buchneri 1 × 10^9^ cfu/g, and *pediococcus* pentosaceus 1 × 10^9^ cfu/g *Lactobacillus* kimchi 1 × 10^9^ cfu/g, these data were produced by Xi'An Jushengyuan Biotechnology Co., Ltd., the pickled vegetable lactic acid bacteria 1 × 10^9^ cfu/g, which were produced by Beijing Chuanxiu International Trade Co., Ltd., the usage of lactic acid bacteria is determined according to the instructions provided by each manufacturer (Zi et al., 2021).

### Preparations before silage

Fresh alfalfa harvested from saline and non-saline lands is cut to about 2 cm without being dried, and the moisture content is controlled at around 80%. The composition of the alfalfa raw materials is shown in Table 1. Non-saline alfalfa without additives (HNS-CK), non-saline alfalfa with compound lactic acid bacteria (*Lactobacillus* plantarum + *Lactobacillus* buchneri + *Pediococcus* pentosaceus + *Lactobacillus* kimchi, HNS-L4), saline alfalfa with compound lactic acid bacteria (*Lactobacillus* plantarum + *Lactobacillus* buchneri + *Pediococcus* pentosaceus + *Lactobacillus* kimchi, HS-L4), saline alfalfa without additives (HS-CK), after fully mixing, load into polyethylene vacuum packaging bags, which specification is 20 × 25 cm, each bag filled with 200 g, and stored at room temperature. Each treatment has 3 replicates. On the 60^th^ day of silage, the nutritional quality, fermentation quality, and microbial diversity of the silage feed are tested (Chen et al., 2020; Cheng and Lin, 2018; Fu et al., 2022; Wilkinson and Davies, 2013).

**Table 1:** Chemical composition of alfalfa raw materials.

**Alfalfa**	**DM (%FM)**	**CP (%DM)**	**NDF (%DM)**	**ADF (%DM)**	**WSC (%DM)**
Saline-alkali soil	28.6	20.24	40.86	32.96	4.9
Non-saline-alkali soil	28.6	22.01	39.21	31.74	4.1

FM, Fresh alfalfa; DM, Dry matter; CP, Crude protein; NDF, Neutral detergent fiber; ADF, Acid detergent fiber; WSC, Water soluble carbohydrate. The same below.

### Test index determination

#### Silage nutritional quality determination

The silage samples are dried in a 65°C oven until constant weight to measure the dry matter (DM) content 9, and the water soluble carbohydrate (WSC) content is determined by anthrone-sulfuric acid colorimetry. Crude protein (CP) detection uses the Kjeldahl method, neutral detergent fiber (NDF) and acid detergent fiber (ADF) detection uses the Van Soest washing fiber method, and specific detection can refer to Feed Analysis and Feed Quality Detection Technology (Wang, 2011; Zhang, 2007; Chen, 2019; Zhang and Han, 2010).

#### Silage fermentation quality determination

The pH is measured with a portable pH meter HORIBA B-712 type; the contents of lactic acid (LA), acetic acid (AA), propionic acid (PA), and butyric acid (BA) are detected by high-performance liquid chromatography, with a mobile phase of 3 mmol/L perchloric acid, a flow rate of 1 mL/min, a column temperature of 50°C, a detection wavelength of 210 nm, and an injection volume of 5 μL (Han et al., 2003). Ammonia Nitrogen/Total Nitrogen (AN/TN) was determined by colorimetric method (Guan et al., 2020).

### Microbial sequencing

After 60 days of silage, open the bag and mix the samples evenly. Use the E.Z.N.A.^®^ Soil kit Omega, USA to extract the total DNA from the silage feed microorganisms and detect the DNA purity, concentration NanoDrop2000, and quality. Perform PCR amplification on the V3-V4 variable region of the 16S rRNA gene, purify the PCR products with a gel kit AxyPrepDNA, elute with Tris_HCI buffer, and detect the PCR products with 2% agarose gel electrophoresis; use the QuantiFluorTM-ST blue fluorescent quantitative system Promega, USA for quantification, construct the Miseq library with the Tru Seq TM DNA Sample Prep Kit. Miseq instrument sequencing. Microbial species classification analysis is performed using the Qiime platform (http://qiime.org/install/index.html), Alpha diversity analysis is performed using Mothur software (https://www.mothur.org/wiki/Download_mothur), Operational Taxonomic Units (OTU) statistical analysis is performed using Usearch (https://www.drive5.com/usearch/) (Guan et al., 2020, 2018; Zhang, 2016).

### Data statistics and analysis

Data analysis and processing are performed using Excel 2021 and IBM SPSS Statistics27. Conduct an analysis of variance (ANOVA) with a single factor. Perform multiple comparisons using the Duncan method, and present the results as “mean ± standard error.” A *P*-value < 0.05 indicates a significant difference. Plot OUT cluster graphs and the principal coordinate analysis (PCoA) graphs, inter-group difference graphs are plotted using R language version 3.3.1, Anosim test is performed on the PCoA results, Kruskal-Wallis test is performed on inter-group differences.

## Results and discussion

### Characteristics of fresh materials before ensiling

The nutritional composition of alfalfa before ensiling is shown in Table 1, and the basic conditions of saline-alkali soil are shown in Table 2. The crude protein content of alfalfa in saline-alkali and non-saline-alkali soils is as high as 20.24% and 22.01% respectively, with higher contents of neutral detergent fiber and acid detergent fiber, and lower values of soluble carbohydrates, which are 4.9% and 4.1% respectively.

**Table 2:** Soil basal conditions in saline-alkali soil.

**Index**	**Salt content (%)**	**Na^+^ (mg/kg)**	**Cl^−^(mg/kg)**	**Organic matter (%)**	**pH**
Non-saline-alkali soil	0.079	0.127	0.041	10.885	7.043
Saline-alkali soil	0.304	0.332	0.138	14.815	8.51

### Effects of different treatments on the nutritional quality of high moisture alfalfa silage

The effects of different compound lactic acid bacteria additives on the nutritional and fermentation quality of high moisture alfalfa silage are shown in Table 3. After 60 days of silage, the contents of DM, CP, and relative feeding value (RFV) in the HNS-L4 and HS-L4 groups were significantly higher than those in the HNS-CK and HS-CK groups (*P* < 0.05); the highest CP content was 18.90% in the HS-L4 group, and the lowest CP content was 16.43% in the HNS-CK group; the highest RFV was 167.00% in the HNS-L4 group; the contents of ADF and NDF in the HNS-L4 and HS-L4 groups were significantly lower than those in the HNS-CK and HS-CK groups (*P* < 0.05), the lowest ADF and NDF contents were 38.43% and 29.39% respectively in the HNS-L4 group. In summary, compound lactic acid bacteria additives can improve the nutritional quality of high moisture saline and non-saline alfalfa silage, and the nutritional quality of non-saline alfalfa is better than that of saline alfalfa.

**Table 3:** Effect of compound lactic acid bacteria L4 on the quality of alfalfa silage.

	**Non-saline-alkali soil**		**Saline-alkali soil**		
**Index**	**HNS-CK**	**HNS-L4**	**HS-CK**	**HS-L4**	***p***
DM(%FM)	25.41 ± 0.10^b^	27.71 ± 0.21^a^	24.16 ± 0.08^c^	27.58 ± 0.20^a^	< 0.001
CP(%DM)	16.43 ± 0.09^b^	18.83 ± 0.12^a^	16.60 ± 0.25^b^	18.90 ± 0.12^a^	< 0.001
WSC(%DM)	0.72 ± 0.01^c^	0.58 ± 0.03^c^	1.80 ± 0.06^a^	1.33 ± 0.07^b^	< 0.001
NDF(%DM)	39.73 ± 0.11^b^	38.43 ± 0.17^c^	41.24 ± 0.57^a^	39.20 ± 0.05^c^	< 0.001
ADF(%DM)	31.73 ± 0.11^b^	29.39 ± 0.45^c^	33.58 ± 0.24^a^	31.20 ± 0.55^b^	< 0.001
RFV	145.67 ± 0.33^c^	167.00 ± 1.00^a^	134.33 ± 2.40^d^	154.33 ± 2.60^b^	< 0.001
pH	5.98 ± 0.04^b^	4.34 ± 0.02^d^	6.48 ± 0.04^a^	4.72 ± 0.05^c^	< 0.001
LA(%DM)	1.58 ± 0.04^c^	3.35 ± 0.06^a^	0.69 ± 0.10^d^	2.54 ± 0.21^b^	< 0.001
AA(%DM)	1.19 ± 0.07^a^	1.10 ± 0.06^a^	1.27 ± 0.36^a^	0.82 ± 0.22^a^	0.515
PA(%DM)	ND	ND	0.10 ± 0.02^a^	ND	< 0.001
BA(%DM)	ND	ND	0.36 ± 0.04^a^	0.05 ± 0.04^b^	< 0.001
AN/TN(%)	3.95 ± 0.28^a^	2.16 ± 0.27^a^	3.04 ± 0.72^a^	2.19 ± 0.49^a^	0.14

RFV, Relative Feeding Value; LA, Lactic acid; AA, Acetic Acid; PA, Propionic Acid; BA, Butyric Acid; AN/TN, Ammonia Nitrogen/Total Nitrogen. Different letters on the same column indicate significant differences (*P* < 0. 05) . ND indicates no detection.

There were significant differences in pH among all groups (*P* < 0.05), pH values are sorted from the lowest to the highest: HNS-L4 < HS-L4 < HNS-CK < HS-CK, the lowest pH of 4.34 is in the HNS-L4 group and the highest pH of 6.48 is in the HS-CK group; there were significant differences in LA content among all groups (*P* < 0.05), with the order of LA being HNS-L4>HS-L4>HNS-CK>HS-CK, and the highest LA being 3.35 in the HNS-L4 group, the lowest (*P* < 0.05) LA of 0.69 is in the HS-CK group; there were no significant differences in AA and Ammonia Nitrogen/Total Nitrogen (AN/TN) content among all groups (*P* < 0.05), the lowest AN/TN content being 2.16% in the HNS-L4 group. In summary, compound lactic acid bacteria additives can increase the lactic acid content and lower the pH in high moisture saline and non-saline conditions, but the compound lactic acid bacteria groups can only lower the AN/TN content without significant differences compared to the control groups (*P* < 0.05).

### Alfalfa microbial diversity

#### Microbial diversity analysis and dilution curve analysis

Alpha diversity can analyze species evenness Shannon index and Simpson index and species richness Ace index and Chao index within a certain area or system. The Coverage index of all groups was >0.99, indicating that the sequencing results could cover the vast majority of microbial information in the samples. The Shannon index and Simpson index reflect the species richness and evenness in a specific environment and region, with a higher Shannon index indicating higher species richness, and the higher the Simpson index, the greater the evenness of species within the community, but the species richness may be lower. The Chao index and Ace index can represent the estimated number of species, with higher indices indicating higher species richness.

The microbial diversity index of high moisture alfalfa silage with compound lactic acid bacteria additives is shown in Table 4. The Coverage index of all groups was >0.99, indicating that the experimental results could represent the vast majority of microbial information in high moisture alfalfa silage. The Shannon index in the HNS-L4 and HS-L4 groups was significantly lower than that in the HNS-CK and HS-CK groups (*P* < 0.05); the Simpson index in the HNS-L4 and HS-L4 groups was significantly higher than that in the HNS-CK and HS-CK groups (*P* < 0.05); the Chao index and Ace index in the HS-L4 group were significantly lower than those in the HS-CK group (*P* < 0.05), while the Chao index and Ace index in the HNS-L4 group were not significantly different from those in the HNS-CK group (*P* > 0.05). In summary, compound lactic acid bacteria additives can reduce the microbial diversity of high moisture saline and non-saline alfalfa.

**Table 4:** Microbial α diversity index of alfalfa silage with high moisture.

	**Non-saline alfalfa**		**Saline land alfalfa**		
**Index**	**HNS-CK**	**HNS-L4**	**HS-CK**	**HS-L4**	***p***
Shannon index	2.57 ± 0.09^a^	2.03 ± 0.10^b^	2.43 ± 0.03^a^	0.75 ± 0.08^c^	*p < * 0.01
Simpson index	0.14 ± 0.02^c^	0.30 ± 0.03^b^	0.17 ± 0.01^c^	0.69 ± 0.03^a^	*p < * 0.01
Ace index	257.89 ± 22.17^ab^	295.10 ± 60.81^a^	292.85 ± 10.70^a^	168.74 ± 13.37^b^	-
Chao index	243.27 ± 19.69^a^	278.97 ± 53.45^a^	265.60 ± 5.98^a^	147.88 ± 4.30^b^	*p < * 0.05
Coverage index	0.99 ± 0.00^a^	0.99 ± 0.00^a^	0.99 ± 0.00^a^	0.99 ± 0.00^a^	-

RFV, Relative Feeding Value; LA, Lactic acid; AA, Acetic Acid; PA, Propionic Acid; BA, Butyric Acid; AN/TN, Ammonia Nitrogen/Total Nitrogen. Different letters on the same column indicate significant differences (*P* < 0. 05) . ND indicates no detection.

**Figure 1:**
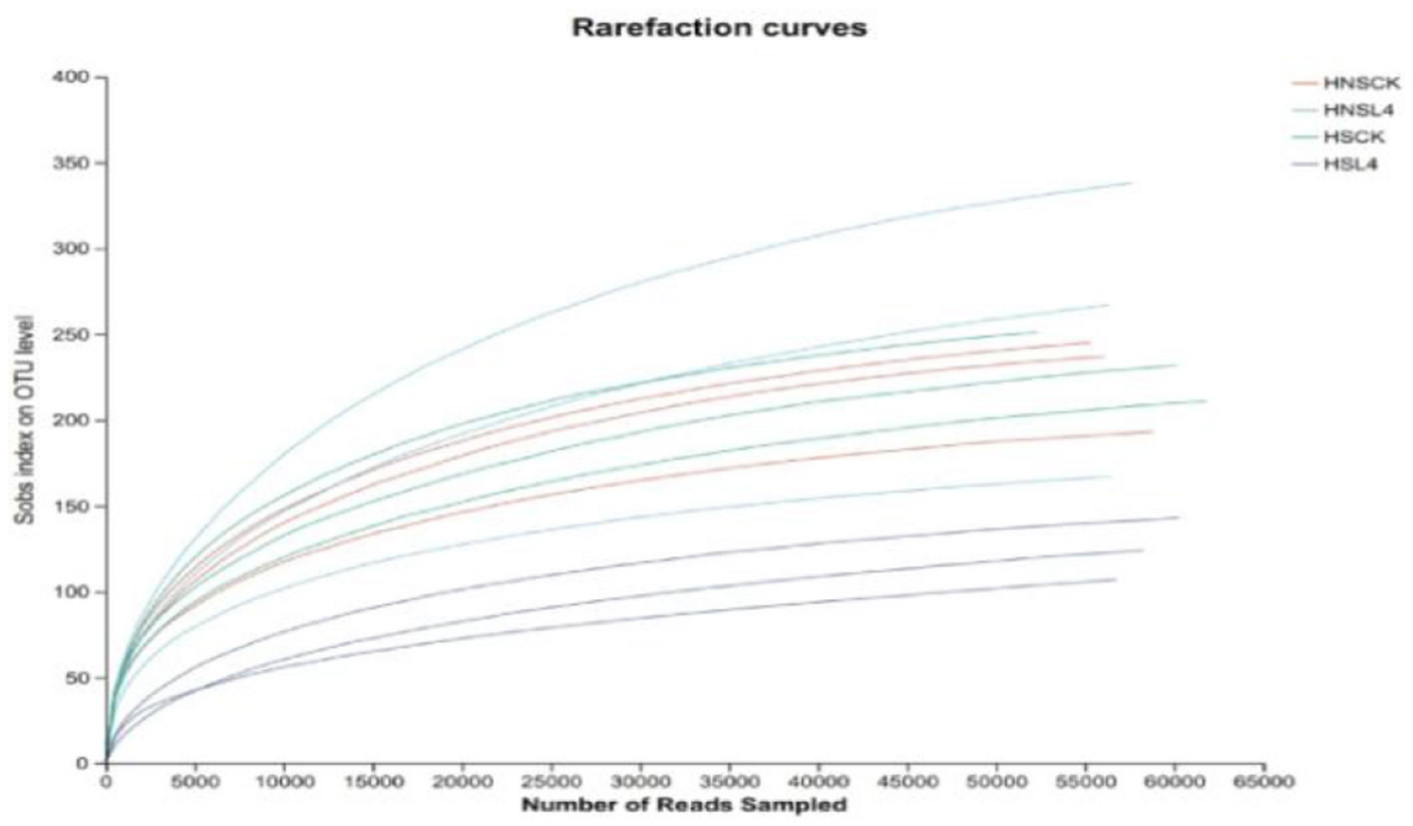
Dilution curve of alfalfa microbial community for 60 days of silage.

As the sequencing volume increases, the dilution curves of alfalfa under different moisture levels tend to flatten, indicating that the experimental results are relatively complete for alfalfa microbial sequencing, and the data can represent the requirements for bacterial community analysis, Figure 1.

#### Microbial community venn diagram

**Figure 2:**
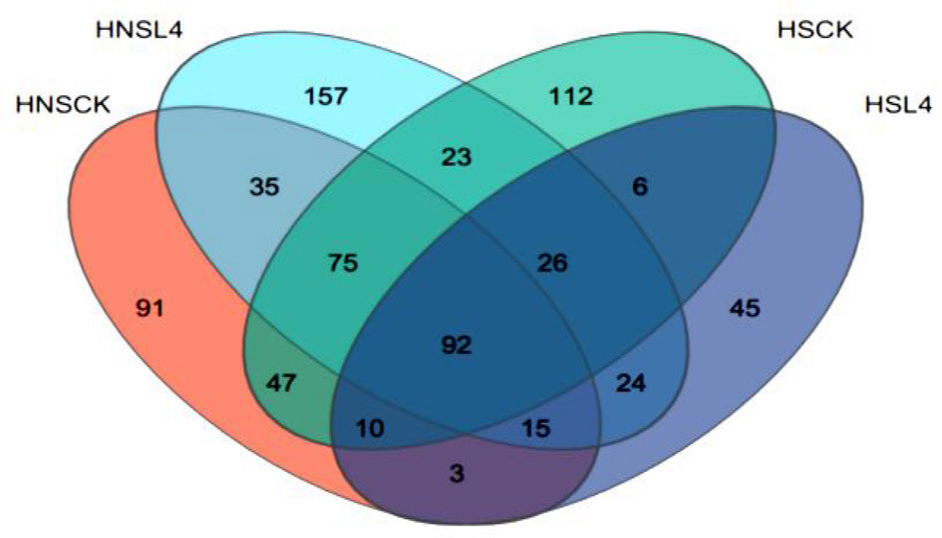
Venn diagram of alfalfa microbial diversity at 60 days of silage.

High-throughput sequencing was performed on the DNA of the alfalfa microbial community. Figure 2 is the Venn diagram of the bacterial community of alfalfa silage for 60 days. As can be seen from the figure, there are differences in the number of OTUs of bacteria adhering to the surface of alfalfa. The number of OTUs for groups HNS-CK, HNS-L4, HS-CK, and HS-L4 are 109, 133, 114, and 88, respectively. After treatment with complex lactic acid bacteria, the HS-L4 group has the fewest number of bacterial species at 88. The number of unique OTUs for groups HNS-CK, HNS-L4, HS-CK, and HS-L4 are 16, 31, 21, and 14, respectively, with the HNS-L4 group and HNS-CK group having the most shared OTUs at 81. In summary, adding compound lactic acid bacteria changes the number of unique microorganisms in silage alfalfa.

### Beta diversity analysis of microbial communities

**Figure 3:**
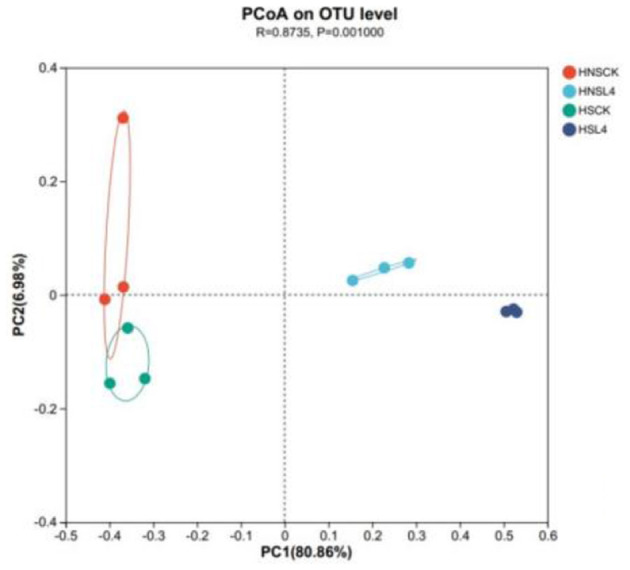
Analysis of alfalfa bacteria principal coordinates based on 60-day silage at OTU level.

As shown in Figure 3, PCoA of bacteria in alfalfa silage for 60 days under high moisture conditions, the experimental groups were divided into four different clusters, namely HNS-CK, HS-CK, HNS-L4, and HS-L4 groups. There is a clear separation between the HNS-L4 and HS-L4 groups and the CK group, while there is no clear separation between the HNS-CK and HS-CK groups, indicating that the lactic acid bacteria preparation has a significant impact on the composition of the bacterial community.

### Microbial community composition

**Figure 4:**
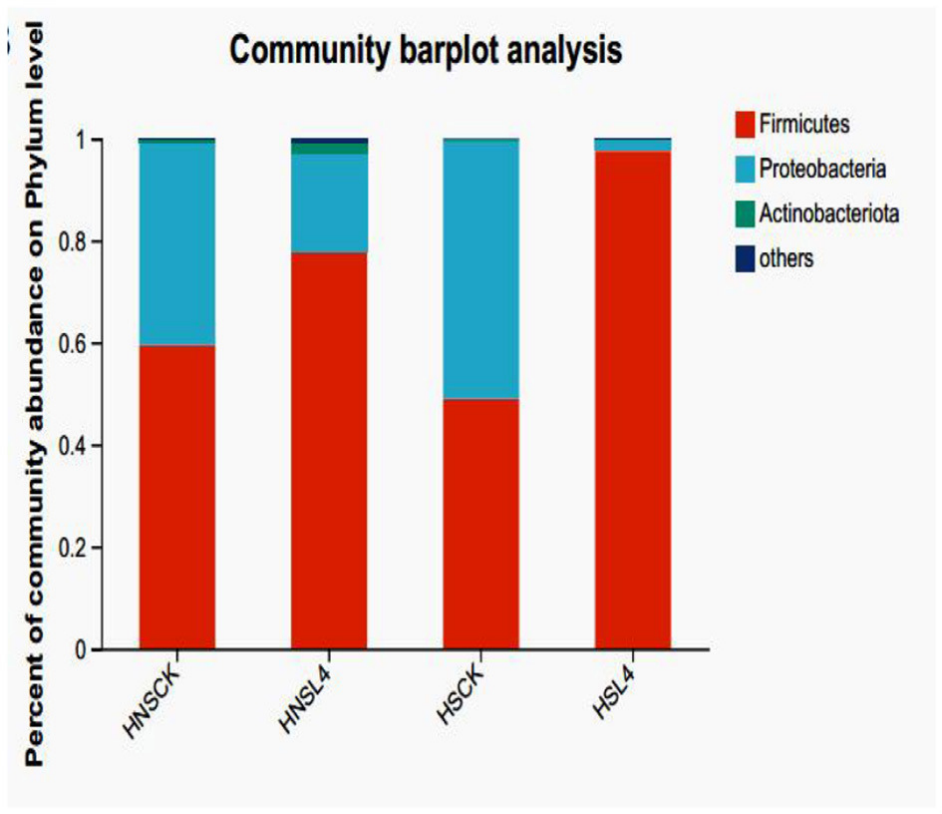
Bacterial community composition of alfalfa silage at the phylum level for 60 day.

The microbial composition of alfalfa after silage at the phylum level is shown in Figure 4 shows the bacterial community composition at the phylum level of alfalfa after 60 days of silage. The dominant phylum in the HS-CK group is Proteobacteria, while the dominant phylum in the other groups is Firmicutes. Compared with the control group, the predominant microorganisms are from the phylum Firmicutes.

**Figure 5:**
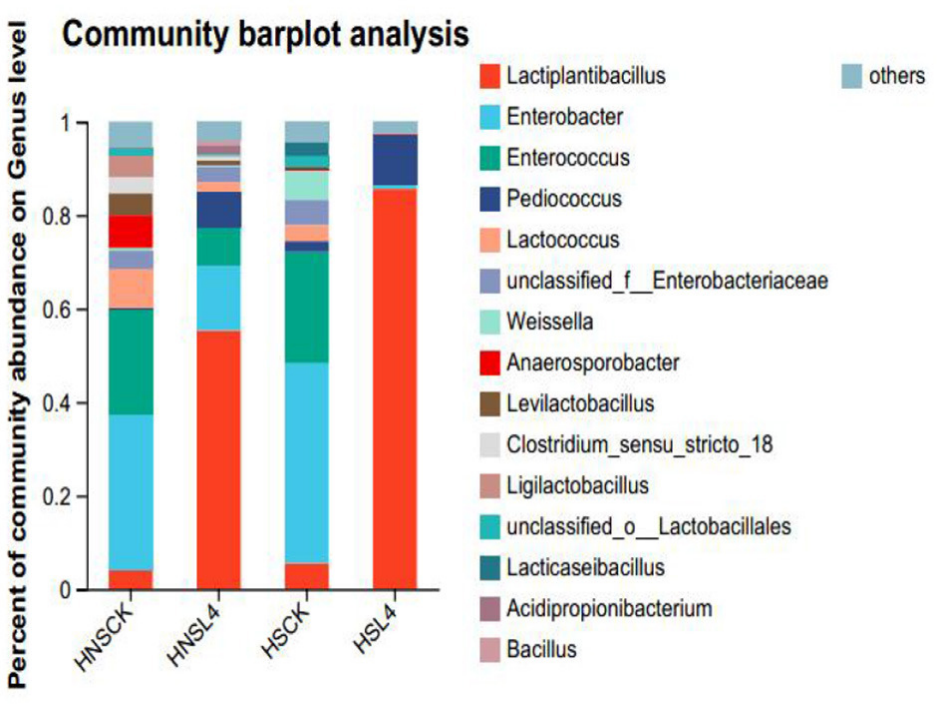
Bacterial community composition of alfalfa silage at the genus level.

The microbial composition of alfalfa after silage at the genus level is shown in Figure 5. Figure 5 shows the bacterial community composition at the genus level of alfalfa silage after 60 days under high moisture conditions. The dominant genus in both the HNS-CK and HS-CK groups was Enterobacter, with relative abundances of 33.37% and 42.70%, respectively. The dominant bacteria in both the HNS-CK and HS-CK groups are *Enterobacteriaceae*, with relative abundances of 33.37% and 42.70%, respectively; the dominant bacteria in both the HNS-L4 and HS-L4 groups are *Lactobacillus*, with relative abundances of 55.34% and 85.62%, respectively. In summary, after additive treatment, the dominant bacteria in the high moisture alfalfa group also changed to *Lactobacillus*.

### Species-level differences in silage microbial communities

**Figure 6:**
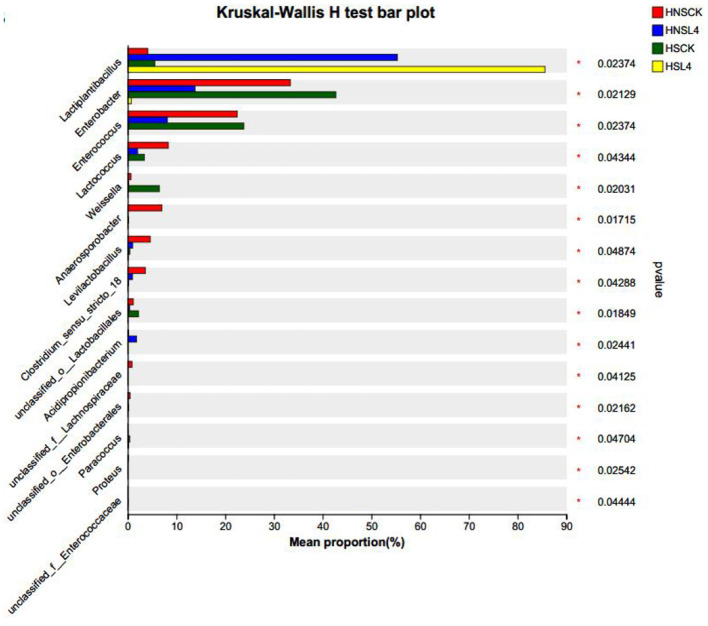
Microbial differences at the genus level in 60-day silage of high-water alfalfa.

To further understand the impact of complex lactic acid bacteria on the microbial community of alfalfa silage, analysis was conducted on the top 15 genera with the highest abundance in each treatment group. The results indicated significant differences in the abundance of bacterial groups among the groups. As shown in Figure 6, which illustrates the microbial differences at the genus level in 60-day silage of high-moisture alfalfa, compared with HNS-CK, the lactic acid bacteria in all groups significantly increased (*P* < 0.05); compared with HS-L4, the lactic acid bacteria in all groups significantly decreased (*P* < 0.05), and the *Enterobacteriaceae* spp significantly increased *(P* < 0.05); compared with HNS-CK, the *Weissella* spp in HS-CK group significantly increased (*P* < 0.05); compared with HNS-L4, the *Enterococcus* and *Lactococcus* spp in HNS-CK and HS-CK groups significantly increased (*P* < 0.05).

### Correlation analysis between silage microbial composition and silage quality at the genus level

**Figure 7:**
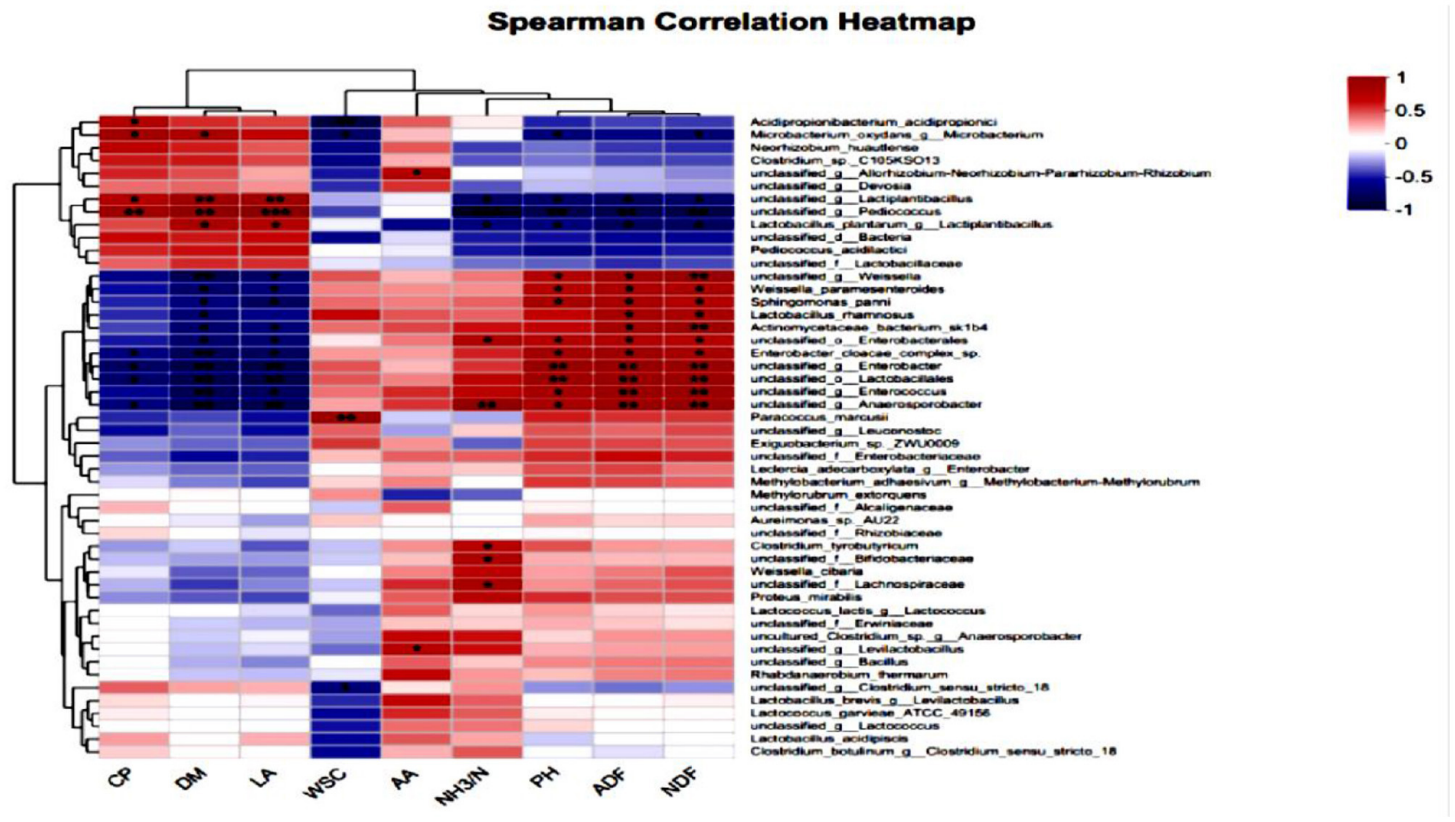
Heatmap analysis of the correlation between alfalfa silage quality and bacterial genus at 60 days of silage.

As shown in Figure 7, which illustrates the correlation heatmap of silage quality and bacterial genera in high-moisture alfalfa after 60 days of silage, the lactic acid bacteria showed a positive correlation with DM and LA, and a negative correlation with pH, ADF, and NDF; the anaerobic spore-forming bacteria, *Weissella* spp, unclassified *Proteobacteria, Enterococcus, Enterobacteriaceae*, and unclassified *Lactobacillus* spp showed a negative correlation with DM and LA, and a positive correlation with pH, ADF, and NDF.

## Discussion

### Alfalfa silage quality in saline and non-saline soils

Nutritional quality is a judgment indicator for the value of forage utilization, with DM mainly referring to the weight of organic matter in silage forage (Geary et al., 2015). In this experiment, the loss rate of DM in all groups treated with compound lactic acid bacteria was lower than that of the control group, and the DM content decreased compared to the raw material. The research results differ from those (Wang et al., 2022), because they added grain concentrate such as corn during alfalfa silage, leading to an increase in the dry matter content of the feed. Studies have shown that after adding bacterial agents to alfalfa silage under different degrees of salinity and alkalinity, as the salt stress increases, there is no significant difference in DM between groups (He et al., 2021), which is consistent with the changes in DM under two different moisture gradients in this experiment.

CP content is an important indicator to evaluating the nutritional value of forage (Guo et al., 2018), and it is also a symbol for evaluating the value of feed. When the pH in the silage environment reaches the level that can inhibit the activity of proteolytic bacteria, the protein in the plant material can be preserved (Luo et al., 2020; Dong et al., 2022). In this experiment, the pH of the compound lactic acid bacteria treatment group after 60 days of silage was significantly lower than that of the treatment group without additives, and the CP content was significantly higher than that of the treatment group without additives, indicating that after adding complex lactic acid bacteria, the activity of proteolytic enzymes in the treatment group was effectively inhibited. After adding fermentation promoters to non-saline-alkali and saline-alkali soils respectively, the CP content of the non-saline-alkali soil control group was significantly lower than that of the saline-alkali soil control group, possibly because more spoilage bacteria proliferated during the silage process in non-saline-alkali soil, thus degrading more protein in the forage (Hou, 2021; Junges et al., 2017; Fu et al., 2013). The saline-alkali soil control group, due to the larger content of Na^+^, inhibited the reproduction of spoilage bacteria and reduced the amount of protein decomposition (Robert et al., 2000; Hu, 2021; Hughes, 1971; Lu et al., 2021). The research results indicate that compared with the control group, adding lactic acid bacteria and sucrose to silage can significantly increase the crude protein content, which is consistent with the results of this experiment, and the CP content of the compound lactic acid bacteria group in saline-alkali soil is greater than that in non-saline-alkali soil under both moisture conditions (Wang et al., 2018; Bao et al., 2023; Hou et al., 2017).

The evaluation standard for feed quality can also be determined by the level of NDF content in silage feed, and its grading standard can reflect whether the concentrate-to-roughage ratio in the diet fed to animals is optimal. The evaluation standard for the energy level in feed can be determined by the level of ADF content in silage feed, and generally, the higher the feeding value of the feed, the lower the content of acid detergent fiber (ADF) (Marshall et al., 1992). Studies have shown that saline-alkali soil will increase the content of ADF and NDF because salt stress will increase the synthesis of crude fiber (Yu et al., 2019), which is consistent with the results of this experiment. In this study, whether before or after silage, the ADF and NDF content of the saline-alkali soil control group and the bacterial agent group were higher than those of the non-saline-alkali soil control group and the bacterial agent group. In the results of this experiment, the WSC content of alfalfa silage in saline-alkali soil was always higher than that in non-saline-alkali soil after silage (Broderick et al., 2000; Zhang et al., 2015), which on the one hand is related to the different raw materials attached to the undesirable microorganisms that can utilize soluble carbohydrates, and on the other hand the relationship between forage and WSC to alleviate salt stress (Li et al., 2010; Ma, 2020; Dong et al., 2021; Luo et al., 2021).

The higher LA, contributes to the lower pH, ending up with the better quality of silage feed. The fermentation products of beneficial bacteria during silage are LA, which is the fundamental guarantee for successful fermentation and safe storage (Ma, 2019; Kung et al., 2003; Tharangani et al., 2022). In this experiment, the main components of the compound lactic acid bacteria were homofermentative lactic acid bacteria, which produce a large amount of lactic acid to quickly lower the pH in the silage environment and establish an acidic environment, so the lactic acid content of compound lactic acid bacteria groups were significantly higher than that of the CK group, and the pH were significantly lower than that of the CK group (Kleinschmit and Kung, 2006; Jayaram et al., 2014; Kung and Ranjit, 2001).

### Effects of different saline-alkali soil alfalfa silage quality and microbial diversity

In the later stage of silage fermentation, lactic acid bacteria will become dominant, the microbial diversity in the later stage of silage will decrease. From Figures 5, 6, it can be seen that the microbial diversity of silage alfalfa is more than that of the additive group. It may be that the spoilage bacteria in the control group were not well controlled during the silage process, so the quality after silage was lower, which is consistent with the results of Wang (2018) and Fan et al. (2023) on the study of silage sorghum, and its microbial diversity also has a similar situation. The Beta diversity of silage bacteria community is positively significantly correlated with the sodium ion content in silage alfalfa, because the different microbial communities in silage forage may be due to the different Na^+^ content in different saline-alkali alfalfa (Lu, 2022). The research results of Jing et al. (2019) indicate that if the ion content is high, it will affect the transmission of electrical signals between microorganisms, inhibit the reproduction and growth of microorganisms, which is consistent with the results of this study. In this experiment, by analyzing the microbial diversity index table, the microbial diversity of silage alfalfa in saline-alkali soil was lower than that in non-saline-alkali soil (Xu et al., 2018; Xu, 2005; Yokoi et al., 2002).

At the phylum level, after 60 days of silage, except for the HS-CK group, the dominant bacteria in each group were the Firmicutes phylum, followed by the Proteobacteria phylum. The number of Firmicutes in the additive group under each saline-alkali component was greater than that in the control group. Studies have shown that after 60 days of silage, the dominant bacteria in alfalfa silage feed are the Firmicutes phylum, Proteobacteria phylum, Actinobacteria phylum, and Bacteroidetes phylum, which are consistent with previous reports on alfalfa silage and corn silage (Lei et al., 2015). Among them, the Proteobacteria phylum and the Firmicutes phylum are the largest phylum in alfalfa silage fermentation (Bai et al., 2020), which is consistent with the results of this experiment.

At the genus level, after 60 days of silage, the main lactic acid bacteria that played a role were *Lactobacillus* and *Enterococcus*, which is consistent with the research results of Jacxsens et al. (2003) on the bacteria after silage. In many studies on microbial communities, it has been shown that *Lactobacillus* rapidly proliferates in the late anaerobic environment of silage, reducing pH (Jacxsens et al., 2003; Cai and Kumai, 1994; Mahmood Fashandi et al., 2018), becoming another dominant bacterial group besides *Enterococcus* and *Lactococcus* (Jacxsens et al., 2003; Anjum et al., 2014; Lü et al., 2011; You, 2023; Yu, 2011; Yuan et al., 2020). In the results of this experiment, the Lactobacillus of the group with compound lactic acid bacteria was higher than that of the control group, and the pH of the additive group was lower, and the lactic acid content was higher. Spearman correlation analysis showed that the Lactobacillus was positively correlated with DM and LA quality, and negatively correlated with pH, ADF, and NDF, which also confirmed this point. Studies have shown that the microorganisms of Enterobacteriaceae continuously consume some nutrients during the fermentation process of silage feed to promote their own growth and reproduction (Bijelic et al., 2015; Hu, 2021). In this experiment, the richness of *Enterobacteriaceae* in the additive group of silage alfalfa was significantly lower than that in the control group of silage alfalfa, indicating that the use of complex lactic acid bacteria reduced the loss of nutrients to some extent (Mansfield and Kuldau, 2007). Spearman correlation analysis indicated that *Enterobacteriaceae* was negatively correlated with DM and LA quality, and positively correlated with pH, ADF, and NDF.

In the results of this experiment, the HNS-CK and HS-CK groups both detected the genus *Weissella* spp, and HS-CK detected *Pseudomonas* spp. After 60 days of silage, the CP and WSC of each group were reduced to varying degrees compared to the raw material. Lu (2022) studies have shown that after 60 days of ensiling, the content of crude protein (CP) and water-soluble carbohydrates (WSC) in saline-alkali alfalfa decreases, which is due to the presence of contaminants such as *Enterobacter, Pseudomonas, and Weissella* spp. These contaminants utilize nutritional components like WSC (Zhang et al., 2020), leading to increased consumption of nutrients during ensiling fermentation (Cai et al., 1999), and a decline in nutritional quality. These contaminants not only consume WSC but also reduce the efficiency of its utilization (Blajman and Vinderola, 2020).

## Conclusion

The results of ensiling alfalfa from non-saline-alkali and saline-alkali lands without additives indicate that ensiling alfalfa from saline-alkali land has the worst effect. However, after treatment with complex lactic acid bacteria, the quality of ensiling in all groups improved, and the quality of alfalfa from non-saline-alkali land was better than that from saline-alkali land. Lactic acid bacteria, to some extent, improved the microbial community structure of ensiled alfalfa. After treatment with lactic acid bacteria, microbial diversity decreased and species were reduced. At the genus level, the abundance of *Lactobacillus* increased; at the phylum level, the Firmicutes bacteria dominated in the compound lactic acid bacteria group.

## Data Availability

The original contributions presented in the study are included in the article/Supplementary material, further inquiries can be directed to the corresponding author.
